# Identification of the Rostral Migratory Stream in the Canine and Feline Brain

**DOI:** 10.1371/journal.pone.0036016

**Published:** 2012-05-11

**Authors:** Saafan Z. Malik, Melissa Lewis, Alison Isaacs, Mark Haskins, Thomas Van Winkle, Charles H. Vite, Deborah J. Watson

**Affiliations:** 1 Department of Neurosurgery, School of Medicine, University of Pennsylvania, Philadelphia, Pennsylvania, United States of America; 2 Department of Clinical Studies, University of Pennsylvania, Philadelphia, Pennsylvania, United States of America; 3 Department of Pathobiology, School of Veterinary Medicine, University of Pennsylvania, Philadelphia, Pennsylvania, United States of America; Universitätsklinikum Carl Gustav Carus an der Technischen Universität Dresden, Germany

## Abstract

In the adult rodent brain, neural progenitor cells migrate from the subventricular zone of the lateral ventricle towards the olfactory bulb in a track known as the rostral migratory stream (RMS). To facilitate the study of neural progenitor cells and stem cell therapy in large animal models of CNS disease, we now report the location and characteristics of the normal canine and feline RMS. The RMS was found in Nissl-stained sagittal sections of adult canine and feline brains as a prominent, dense, continuous cellular track beginning at the base of the anterior horn of the lateral ventricle, curving around the head of the caudate nucleus and continuing laterally and ventrally to the olfactory peduncle before entering the olfactory tract and bulb. To determine if cells in the RMS were proliferating, the thymidine analog 5-bromo-2-deoxyuridine (BrdU) was administered and detected by immunostaining. BrdU-immunoreactive cells were present throughout this track. The RMS was also immunoreactive for markers of proliferating cells, progenitor cells and immature neurons (Ki-67 and doublecortin), but not for NeuN, a marker of mature neurons. Luxol fast blue and CNPase staining indicated that myelin is closely apposed to the RMS along much of its length and may provide guidance cues for the migrating cells. Identification and characterization of the RMS in canine and feline brain will facilitate studies of neural progenitor cell biology and migration in large animal models of neurologic disease.

## Introduction

Accumulating evidence in experimental models has shown that CNS damage alters the migration patterns of endogenous neural progenitor cells (eNPCs). Reports from numerous laboratories have shown that, in rodents, eNPCs divert towards a wide range of brain pathologies, a process termed pathotropic migration, ectopic migration or homing (reviewed in [1]). These rodent disease models include cerebral infarction, seizures, tumors, demyelination, and neurodegeneration which are induced genetically, surgically or chemically.

In dogs and cats, however, these diseases occur spontaneously just as they do in humans. Naturally-occuring diseases in cats and dogs have a significant advantage for translational research in that they frequently demonstrate the same clinical signs and pathophysiology as the diseases in humans (see [2]). As an example, naturally-occuring canine brain tumors closely replicate many critical features of human brain tumors, including incidence, clinical findings, histopathology, imaging, gene expression profiles, and cancer stem cells [3]–[11]. Importantly, the development of these tumors (and potential therapies) can be tracked over months compared to just a few weeks in rodent models.

In addition, the study of large animals offers a number of other advantages for translational research to bridge the gap between rodents and humans. The physical organization of the large animal brain is more similar to the human brain than is the murine brain. The nervous systems of both the dog and cat have been well characterized anatomically, physiologically, and clinically [12]–[15]. In contrast to rodent models, the greater similarities of these large animal disease models to the human conditions may improve the possibilities for success in translating potential therapies into humans. Further, studies of animal models other than mice could provide very useful information about evolution of this important migratory stream in the adult brain and the possible function of postnatal neuronal replacement.

The rostral migratory stream of eNPCs has not yet been identified in canines or felines. Characterization, mapping and measuring the RMS in normal dogs and cats is an important step to lay the foundation for future studies of eNPC migration and function in translational models of CNS disease. Both laboratory animals and companion animals offer greater flexibility and more opportunities for research studies including: (1) Scale-up testing of promising therapeutic agents identified in rodent experiments; (2) Compared to rodents, longer longitudinal monitoring of therapeutic effects with biomarkers, biopsies and imaging, including after radiation, chemotherapy and/or surgery; and (3) Compared to humans, canine and feline brains are more readily available post-mortem for histopathological assessment. Indeed, these advantages have spurred calls for more clinical trials to be performed in companion animals [16]–[18].

In this report, we describe the anatomical route and dimensions of the normal canine and feline RMS and an initial characterization of the cellular phenotypes.

## Results

**Figure 1:**
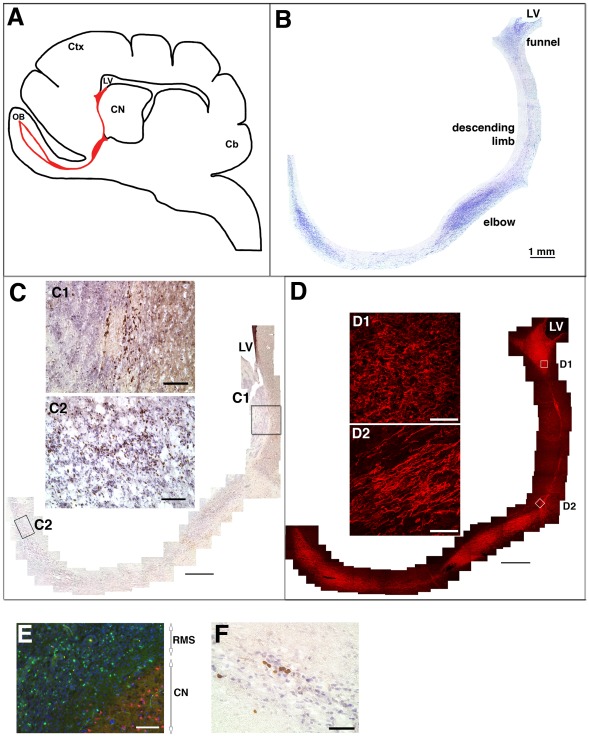
Rostral migratory stream in the dog brain. (A) Schematic of a sagittal view of a canine brain. The red line indicates the location of the RMS in relationship to the anterior horm of the lateral ventricle (LV), caudate nucleus (CN), olfactory bulb (OB), cortex (Ctx) and cerebellum (Cb). (B) Nissl staining showing the orientation and nomenclature of the canine RMS. (C) anti-BrdU immunostaining in brown with hematoxylin counterstain in purple. C1 shows BrdU staining at the boundary of the white matter and caudate nucleus in the descending limb; C2 shows BrdU staining in the olfactory peduncle/rostral limb. (D) anti-Dcx immunostaining. D1 shows morphology of cells in the funnel; D2 shows leading and trailing processes of migrating cells in the descending limb. (E) Immunoreactivity for BrdU (green, in the RMS) and NeuN (red, in the CN) does not overlap in the descending limb. Section is counterstained with DAPI (blue). (F) BrdU immunostaining (brown) in the olfactory peduncle in tissue from dog 5, analyzed at 6 hr after a single 75 mg/kg i.v., indicating that BrdU is taken up by dividing cells all along the RMS. Scale bar in B, C, D: 1 mm. Scale bars in C1, C2, E: 100 microns. Scale bars in D1, D2, F: 50 microns. Please view the figures on a computer monitor for accurate RGB color representation.

In the dog brain, Nissl staining of sagittal sections revealed a prominent, dense, continuous track of cells, beginning at the base of the anterior horn of the lateral ventricle (LV) dorsal to the caudate nucleus, curving around the rostral portion of the head of the caudate nucleus and continuing ventrally to the olfactory peduncle and olfactory bulb (Fig. 1A–B). When posssible, Nissl-stained sections were used to measure the length of the RMS and its width at the widest point of the descending limb. Sections covering the entire RMS were of course not available in those subjects in which the olfactory bulb had not been captured. However, in sections from a 5 year old Springer/Schipperke that did contain the entire RMS and the olfactory bulb, the distance from the rostral tip of the LV to the rostral end of the olfactory ventricle was 31.5 mm and the widest point of the descending limb was 308 microns. Other dogs had shorter but wider RMS tracts. The five subjects in this group prevent firm conclusions regarding the effect of age or species, but our data do provide a preliminary indication of the scale and location of the RMS (Table 1, Fig. 1). A 3D view of the RMS in the Springer-Schipperke is presented in Movie S1. This movie demonstrates that the RMS is not contained in one sagittal plane; rather, it bends laterally as it progresses down along the descending limb, then back to the original medial plane as it approaches the olfactory bulb.

**Table 1:** Experimental Subjects.

			BrdU dose	No. Injections	Time of perfusion after final	RMS Length	Hemisphere Length	RMS Length	DL Widest Sagittal
Subject	Age	Breed	(mg/kg)	(1 per day)	BrdU dose	(mm)	(cm)	(normalized)	Dimension (µm)
Cat 1	1 yr	domestic shorthair	75 mg/kg	1	6 hr	15.1	4.9	3.1	173
Cat 2	1 yr 8 mo	domestic shorthair	25 mg/kg	5	24 hr	11.6	nd	nd	381
Cat 3	1 yr 10 mo	domestic shorthair	25 mg/kg	5	24 hr	14.2	4.9	2.9	163
Cat 4	2 yr 9 mo	domestic shorthair	25 mg/kg	5	24 hr	15	4.7	3.2	659
Cat 5	3 yr 7 mo	domestic shorthair	25 mg/kg	5	24 hr	10.1	4.6	2.2	177
Dog 1	1 yr	Keeshond	75 mg/kg	4	24 hr	20.2	8.2	2.5	867
Dog 2	1 yr 3 mo	Lab/Beagle	25 mg/kg	5	24 hr	nd	9.1	nn	851
Dog 3	2 yr 5 mo	Keeshond	75 mg/kg	4	24 hr	nd	9.1	nn	847
Dog 4	5 yr 3 mo	Springer/Schipperke	25 mg/kg	5	24 hr	31.5	8.3	3.8	308
Dog 5	5 yr 6 mo	Corgi/Beagle	75 mg/kg	1	6 hr	nd	7.2	nn	367

nd, not determined (too few slides to cover entire track); nn, not normalized.

To label CNS cells in S phase, we administered the nucleotide analog BrdU intravenously (for timetable and doses see Table 1) and examined the brains by immunostaining for BrdU at 24 hr after the last BrdU injection. In brains from dogs that received multiple doses of BrdU, we found BrdU-immunoreactive cells in the same track identified by Nissl staining. BrdU-positive cells were present in the ventral wall of the anterior horn of the lateral ventricle and dispersed throughout the descending limb and rostral limb into the olfactory peduncle (Fig. 1C). Many of the BrdU-positive cells in the descending limb were located at the boundary between the caudate nucleus and the rostral white matter (see Fig. 1C1) and were generally present as single cells or in closely apposed small groups. These cells were not stained in control sections on which the primary (anti-BrdU) antibody was omitted, nor were they stained in brain sections from dogs that did not receive BrdU (data not shown). BrdU-positive cells in the descending limb adjacent to the caudate mucleus were not double-labeled with the mature neuronal marker NeuN (Fig. 1E).

Cells throughout the entire track expressed doublecortin (Dcx; Fig. 1D), a marker of immature neurons [19]. However, the morphology of the cells differed according to region. Dcx-positive cells in the funnel were tightly clustered and had short processes that were not uniformly oriented (Fig. 1D1). Dcx-positive cells in the descending limb were bipolar, with long leading and trailing processes uniformly oriented along the RMS, indicative of migrating cells (Fig. 1D2).

To determine whether cell division occurred along the entire RMS, or whether dividing cells were present only in the SVZ, a single dose of BrdU was administered and dog 5 was perfused six hours later. BrdU-immunoreactive cells were present along the entire track from the lateral ventricle to the olfactory peduncle (Fig. 1F), indicating that cell division occurs throughout the RMS and not just in the SVZ.

**Figure 2:**
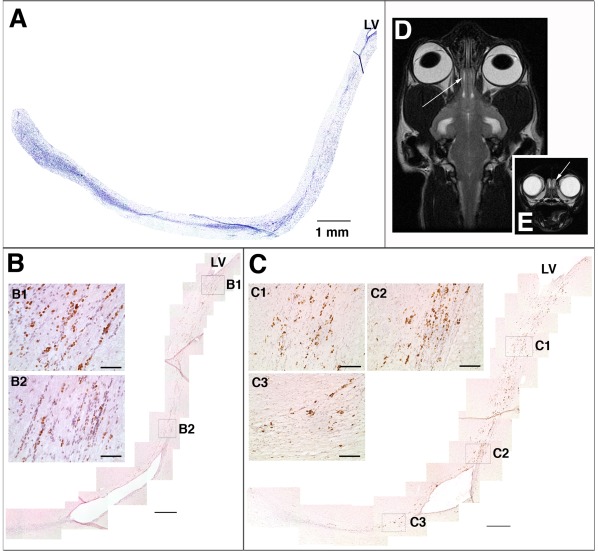
Rostral migratory stream in the cat brain. (A) Nissl staining demonstrating the RMS orientation and location from the anterior horn of the lateral ventricle (LV) to the olfactory bulb (OB). (B) anti-BrdU immunostaining. (C) anti-Ki67 immunostaining, demonstrating the presence of dividing cells along the entire RMS. (D, E) T2-weighted MRI images of a cat head in the dorsal (D) and transverse (E) planes, showing cerebrospinal fluid in the open olfactory ventricles of an adult (5 year old) cat (arrows). Scale bars in main panels A–D: 500 microns. Scale bars in insets B1–2, C1–3∶100 microns.

In the cat brain, an analogous track of cells was identified by Nissl stain (Fig. 2A). These cells were labeled with BrdU (Fig. 2B), and expressed Ki67 (Fig. 2C). Ki67 immunoreactivity was present all along the track, indicating that cell division occurs throughout the track. This is consistent with our finding in the dog that received one dose of BrdU (Fig. 1F). Ki67 staining was present in more cells than BrdU staining, indicating that the BrdU labeled a subset of dividing cells, as expected. In this series of cat brains, the maximum RMS length from the lateral ventricle to the rostral end of the olfactory ventricle was 15.1 mm in the youngest cat (1yo). The RMS length and the brain length were somewhat more uniform in cats then in dogs, likely owing to less variability in size and shape of the brain among adult domestic shorthair cats (Table 1).

We noted that the ventricular extension between the lateral ventricle and the olfactory ventricle was patent in some locations along the tract (Fig. 2B–C), consistent with T2-weighted MRI images of open olfactory ventricles in the cat (Fig. 2D–E).

**Figure 3:**
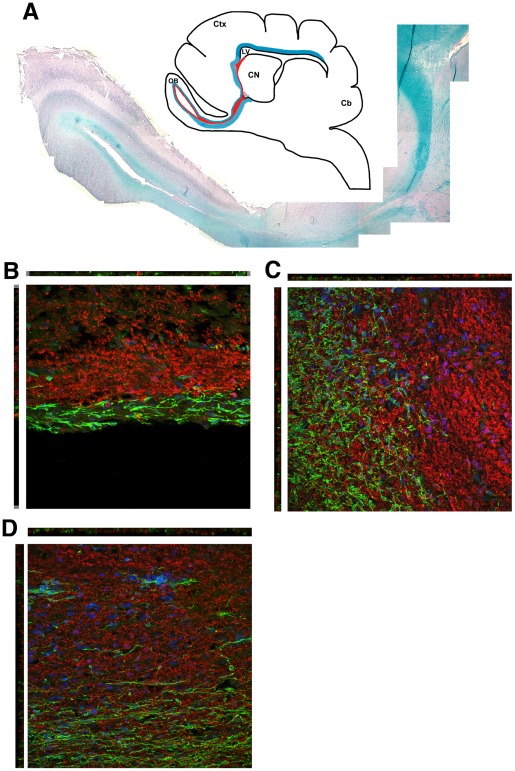
Relationship of white matter and the RMS. (A) Luxol fast blue staining of a saggital section from dog 4. Inset shows the approximate location of the RMS in red and part of the white matter in blue. (B–D) Confocal maximum projection images of CNPase staining (red) and doublecortin staining (green) in the SVZ (B), funnel (C), and olfactory peduncle (D) in dog 1.

In the dog brain, sections in which the white matter was stained with Luxol Fast Blue suggested that myelin is closely apposed to the RMS along its entire length (Fig. 3A and schematic inset). To examine the relationship between oligodendrocytes and neural progenitors, we co-stained sections with Dcx and CNPase (Fig. 3B–D). There was no overlap in the staining pattern of the two markers, indicating that they identify two distinct populations of cells. In the subventricular zone, the two layers are clearly separated with the Dcx-positive layer closer to the lateral ventricle (Fig. 3B). In the funnel area, the two cell populations are intermixed (Fig. 3C) before the Dcx-positive cells proceed ventrally into the descending limb which runs parallel and ventral to the myelin layer. The myelin layer above the funnel, which is part of the corpus callosum, appears to form the dorsal limiting border of the RMS. At the base of the descending limb, the white matter partially provides a caudal boundary for the turn. The RMS then turns rostrally towards the OB where LFB-staining surrounds the track in the OB, again with a clear separation between the CNPase and the Dcx staining (Fig. 3D).

**Table 2:** Primary Antibodies.

Antigen	Species	Supplier	Catalog #	Clone #	Reactivity
Ki-67 (hu)	mouse	DAKO	M7240	MIB-1	Cat
NeuN	mouse	Millipore	MAB377	A60	Dog/Cat
BrdU	rat	Accurate	OBT0030	BU1/75	Dog/Cat
Dcx (hu C-ter)	goat	Santa Cruz	sc–8066	C–18	Dog/Cat
CNPase (hu)	mouse	Sigma	C–5922	11–5B	Dog

## Discussion

We have located and made an initial characterization of the RMS in the canine and feline brain. This track corresponds closely to the reported location and route of the RMS in the human brain but is much longer in proportion to the rest of the brain than in the human. Neural progenitor cells in the adult brain undergo stereotypical patterns of migration from restricted neurogenic niches to specific targets. In the mouse, cells originating in the subventricular zone migrate through the RMS into the olfactory bulb, where they differentiate into mature olfactory inhibitory interneurons [20].

**Figure 4:**
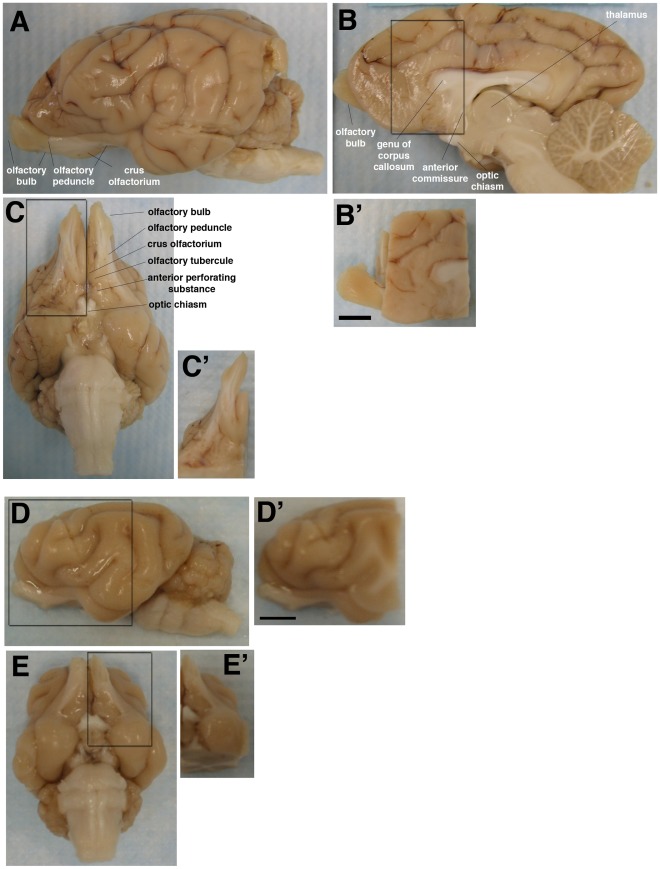
Areas of dog brain (A–C) and cat brain (D–E) embedded for sectioning. (A, D): Lateral view. (C, E): Ventral view. (B): View from the midline. Hemispheres were separated and an ∼40×22×22 mm block (including the olfactory bulb when possible) was isolated from each side (B’–E’). Scale bar in B’ and D’ is 1 cm. Labels are based on the atlas of Singer.

An pathway analogous to the mouse RMS but somewhat more complex has been described in macaque, rhesus and squirrel monkeys and in humans [21]–[25]. We have found that the canine and feline RMS tracts bear some similarities and some differences to the primate and human tracts. In the dog and cat, the RMS comprises a dense track of proliferating cells beginning at the base of the anterior horn of the lateral ventricle dorsal to the caudate nucleus, which curves around the head of the caudate nucleus and turns rostrally, ventrally and somewhat laterally before bending back towards the midline as it reaches the olfactory peduncle (see 3D reconstruction in Movie S1). In the human RMS, the descending limb moves caudally around the base of the caudate nucleus before taking a sharp rostral turn, whereas the DL of the canine/feline track moves rostrally and ventrally without a sharp turn at the base of the caudate nucleus. Another difference is the very large size of the dog funnel, originating in the anterior horn of the lateral ventricle. Also, there is a notable thickening of the RMS at the elbow in the canine brains. The maximum RMS track length in our canine series (31.5 mm in dog D4) is much longer than the estimated length of the RMS in humans in one study (∼17 mm) [25], most likely due to the elongated brain shape and ventricle position in dogs, as well as to the much larger olfactory peduncle and OB in dogs.

There are also some differences in the RMS between cats and dogs. Our results suggest that the funnel in the cat is much smaller than in the dog and the overall track is much thinner, with little to no enlargement at the elbow. The cat RMS was also more uniform in length compared to the dogs, which were of different breeds (see below). As a practical matter, some commercial antibodies react with one species or the other (see Table 2).

The five dogs and five cats used in this study were relatively young and each one was of a different age (see Experimental Procedures and Table 1). A larger number of animals in each age group would be required to assess the effects of age on the overall dimensions of the track. We hypothesize that the SVZ and RMS decrease in size with age, as has been described in the human brain [26]. Generally speaking, such a study of age-related changes would be simpler to do in cats than in dogs. For example, brain size and architecture are generally more uniform in cats than in different breeds of dogs, though both are likely susceptible to age-related changes. In addition, the cats available for this study were all domestic shorthairs, further minimizing variability among the cats. When normalized to total brain length, the length of the RMS is more consistent in cats than in dogs. In this small series of cats, our measurements suggest a decrease in both RMS length and overall brain size after the age of 3 years. Many more cats in each age group would be required to make this a firm conclusion.

Immunostaining for BrdU and Ki-67 indicated that progenitor cell division occurs in the canine and feline brain throughout the entire RMS at least as far as the olfactory peduncle. Similar evidence for progenitor cell division in the RMS has been obtained in human, monkey and rodent brain, by positive staining for dividing cell markers such as BrdU, Ki-67 and/or proliferating cell nuclear antigen (PCNA) [21], [22], [24], [25], [27]. Because dividing cells were present all along the RMS after a single intravenous BrdU injection, we did not use this method to obtain migration data; if and when BrdU-positive cells appeared in the OB, it would not have been possible to determine whether they had migrated to get there. In other words, this method would not distinguish between cells that had migrated all the way from the SVZa versus from an intermediate position in the RMS. Such a kinetic analysis would be facilitated by making a localized injection into the SVZa of a viral vector, BrdU, or another tracer, and analyzing animals at multiple time points.

In the canine and feline brain, as in rodent, monkey and human, the RMS progenitors appear to maintain an immature phenotype as evidenced by positive staining for doublecortin and negative staining for NeuN. As regards the relationship of the myelin and the RMS, a full ultrastructural analysis is required to shed light on whether the white matter provides a boundary for the RMS along all or part of its length, as suggested by the LFB and CNPase results presented here.

In both the dog and cat brain, the ventricular extension between the lateral ventricle and the olfactory ventricle appeared to be patent in some areas. Ciliated ependymal cells were present at the edges of these openings, confirming that the openings were not simply tears in the tissue. A study using casts of the cerebral ventricles in the adult dog suggested that the extension is open along its entire length [28], and this is often seen on T2 weighted MR images of the dog and cat brain (see Fig. 2 for images of cat brain). The olfactory ventricle also persists in the adult rabbit brain [29]. More recently, T2-weighted spin-echo sequences have been used to demonstrate open olfactory bulb ventricles in adult humans [25], [30], although this is a controversial point and may not be observed in all cases [31], [32].

Dogs and cats are increasingly recognized to be important intermediate models to assess therapeutic strategies for naturally-occurring diseases which also occur in human patients. In addition, many induced models of disease have been developed, including spinal cord injury, implanted tumors, brain trauma, demyelination and other neurodenerative diseases [15], [33]–[35]. Some unique resources for studying dog models are available, such as microarray chips and the sequence of the dog genome [36]. In the cat, neuroimaging is arguably more advanced and the brains are more uniform, making sterotactic injection/transplantion simpler and more reproducible. Neural progenitor cells have been isolated and cultured from both the fetal and early postnatal dog and cat brain and are available for transplantation studies in both normal brain and disease models [37]–[42].

In summary, this report identifying, mapping and measuring the RMS in the dog and cat lays the groundwork for future studies to better understand the involvement of eNPCs in the pathogenesis of neurologic disorders.

## Materials and Methods

All procedures involving animals were approved by the University of Pennsylvania Institutional Animal Care and Use Committee and conform to NIH and USDA guidelines. All efforts were made to minimize the number of animals and their suffering. A total of 5 colony-raised cats (1–3.5 yr old) and 5 dogs (1–5.5 yr old) were used for this study (see Table 1). We prepared sagittal sections from canine and feline brain that encompassed the anterior horn of the lateral ventricle, the rostral part of the caudate/putamen, the olfactory tracts and, when available, the olfactory bulbs (see Fig. 4).

### BrdU Administration and Immunohistochemistry

Freshly prepared 5-bromo-2-deoxyuridine (BrdU) in saline (25 or 75 mg/kg i.v.) was administered to each experimental animal (see Table 1). Cat 1 and Dog 5 received 1 dose of BrdU followed by perfusion 6 hr later. All other animals received 1 dose of BrdU per day for 4 or 5 days as noted and were perfused 24 hr after the final dose. Perfusions were performed with heparinized saline followed by formalin. The brains were carefully removed keeping the OBs attached when possible and post-fixed in formalin. After cryoprotection in 30% sucrose, 22X40X20 mm blocks were embedded in Tissue-TEK OCT compound, frozen, and sectioned at 20 microns.

Antigen retrieval was done in 1X Antigen retrieval citra buffer (Biogenex) in a microwave at 40% power for 10 min or in a steamer at 99°C for 15 min. After cooling, H_2_O_2_ treatment and blocking with horse serum, slides were incubated with primary antibody against BrdU (Accurate OBT0030, 1∶1000) overnight at 4°C. The biotinylated secondary antibody was donkey anti rat (Jackson Immunoresearch). ABC reagent (Vector Laboratories) or the DAKOCytomation LSAB2 SystemHRP kit, were applied per manufacturer’s guidelines. The sections treated with ABC reagent were developed with DAB reagent (DAKO), counterstained with hematoxylin (Thermo Scientific), dehydrated, and coverslipped. For immunofluorescence, biotinylated secondary antibodies were detected with streptavidin conjugated to Alexa-594 (Molecular Probes). The mounting medium contained 4′,6-diamidino-2-phenylindole (DAPI) for nuclear visualization.

### Nissl Staining

Every 10th slide was stained with 0.5% cresyl violet for 1 min.

### Other Immunohistochemistry

Slides were rehydrated in PBS. Antigen retrieval was done in 1X Citra Buffer (Biogenex) in a steamer at 99°C for 15 min [43], or in a microwave at 40% power for 10 min. Primary antibodies are listed in Table 2.

Species-specific biotinylated secondary antibodies (Jackson Immunoresearch) were detected with ABC reagents as above. For immunofluorescence, secondary antibodies were biotinylated and detected with streptavidin conjugated to Alexa-488 or were directly conjugated to Alexa-594 (Molecular Probes).

### Image Capture and Analysis

Images were captured with a Nikon E600 Eclipse microscope and SPOT RT3 camera at 1600×1200 pixels and 72 pixels/inch. Each image was proportionally resized in Adobe Photoshop to 480×360 pixels and 320 pixels/inch. Montages were assembled manually or with the Photomerge function. High-power images of Dcx-positive and CNPase-positive cells were captured with a confocal microscope.

## Supporting Information

Movie S1 **This movie represents a medial to lateral series of saggital sections of a dog brain.** Nissl sections are on the left; the correponding track of the RMS is represented in red on the right. The SVZ and LV are at top right of each panel; the olfactory bulb is at the bottom left. The series shows that the RMS is not contained in one plane; rather, the descending limb in particular moves laterally while the funnel and olfactory peduncle remain more medial
(MOV)
